# The impact of primary patella resurfacing on health-related quality of life outcomes and return to sport in total knee arthroplasty (TKA)

**DOI:** 10.1007/s00402-023-04930-x

**Published:** 2023-06-08

**Authors:** Caroline Schatz, Werner Plötz, Johannes Beckmann, Reiner Leidl, Peter Buschner

**Affiliations:** 1 Ludwig-Maximilians-Universität München, LMU Munich School of Management, Institute for Health Economics and Health Care Management, Ludwigstr. 28, 80539 Munich, Germany; 2 Helmholtz Zentrum München, Institute for Health Economics and Health Care Management, Munich, Germany; 3 Environmental Health Center at Helmholtz Munich, Munich, Germany; 4 Krankenhaus Barmherzige Brüder München, Akademisches Lehrkrankenhaus der Technischen Universität München, Munich, Germany; 5 Klinikum Rechts Der Isar, Technical University Munich, Munich, Germany; 6 Orthopedic Praxis Munich-Nymphenburg, Munich, Germany

**Keywords:** Knee, Osteoarthritis, Patella, Patient reported outcome measures, Sports, Health-related quality of life

## Abstract

**Introduction:**

Primary patella resurfacing (PPR) in primary total knee arthroplasty (TKA) is a topic without clear clinical evidence. Using Patient Reported Outcome Measurements (PROM), previous work found TKA patients without PPR to have more pain postoperatively, but little is known whether this may impede patients from returning to their usual leisure sport. This observational study aimed at evaluating the treatment effect of PPR, with PROMs and return to sport (RTS).

**Materials and methods:**

156 primary TKA patients were retrospectively included from August 2019 to November 2020, from a single hospital in Germany. PROMs were measured with the Western Ontario McMaster University Osteoarthritis Index (WOMAC) and the EuroQoL Visual Analog Scale (EQ-VAS), preoperatively and 1 year postoperatively. Leisure sport with three levels of intensity (never, sometimes, regular) were requested. The treatment effect of PPR was evaluated with a difference-in-difference (DiD) approach, with several confounders.

**Results:**

Descriptively, the mean WOMAC total score and the mean WOMAC pain score were postoperatively better with PPR, ( – 4.8 points,  – 1.1 points), then without PPR. The mean improvements of the WOMAC total score were better with PPR ( – 7.8 points). Mean improvements for the WOMAC pain score were also better with PPR ( – 1.2 points). Mean EQ-VAS were postoperatively similar, and the mean improvements were better with PPR (3.4 points). Rate of RTS was 93% for patients with PPR and 95% for patients without PPR. The DiD revealed minor differences in PROMs and RTS, not to result in statistically significant treatment effects.

**Conclusions:**

There was no treatment effect for TKA with PPR, regarding PROMs and RTS, and descriptive differences were below published thresholds for clinical relevance. Rate of RTS was high for all patients, regardless of PPR. For the two endpoint categories, there was no measurable advantage of TKA with PPR over TKA without PPR.

**Supplementary Information:**

The online version contains supplementary material available at 10.1007/s00402-023-04930-x.

## Introduction

Primary patella resurfacing (PPR) in primary total knee arthroplasty (TKA) for patients with osteoarthritis (OA) is a controversially discussed topic in several health care systems around the world. In the US, PPR is conducted regularly, with approximately 90% of all TKA procedures [1], but it is slightly decreasing in recent years. Whereas in Sweden, PPR remains stable within the last decade with less than 5% [26]. In Australia the numbers were increasing, reaching currently more than 75% of all TKA [2]. Due to the numerous risks affiliated with PPR, such as patella fracture or instability, aseptic loosening and osteonecrosis, PPR remains controversial [23]. Recent literature revealed PPR as cost-effective. However, the outcomes were only somewhat different for patients with and without PPR, but the costs were lower, because of reduced reoperations [17, 27].

Previous literature revealed a minor tendency towards more pain for patients without PPR [7, 9, 14] and this might have an impact on the sport habits of patients postoperatively. Patients with more pain may be hindered to return to their usual sport. To the best of our knowledge there is no paper analyzing the return to leisure sport of patients with PPR compared to patients without PPR in primary TKA. Although, in general, patients were often able to return to usual physical activities and sport approximately 1 year after TKA [15]. A review by Hanreich et al. [12] indicated increasing physical activity after primary TKA, with a tendency towards higher benefits for patients below 56 years and towards lower-impact sports, such as walking, biking and swimming. For unicompartmental TKA, more than half of the physically active patients returned to their sport 3 months after surgery, and patients with increased Body Mass Index (BMI) were generally less physically active [19]. Ponzio et al. [21] revealed for TKA that inactive patients improved their activity levels 2 years after surgery. Whereas active patients did not improve their activity levels and had an increased risk of revision [21].

Questionnaires about sport habits and physical activities, especially considering individual sport types, were often not validated and the definitions of sport and physical activity is blurring. Hence, return to sport (RTS), rates of RTS and sport habits were discussed controversially. A thorough use and evaluation of PROMs and the need for further research on RTS was postulated by Degen [8]. This study provides both, an analysis of PROMs and RTS within the same patient cohort. Observational data was analyzed with the aim of revealing whether PPR impacts firstly PROMs, measured with the generic EQ-VAS (EuroQoL Visual Analog Scale) and the disease specific WOMAC, especially the WOMAC pain score. Secondly, the study aimed to analyze RTS as an advancement of both PROMs. Hence, two research questions (RQ) were developed.

RQ1: Could a treatment effect be observed for TKA with PPR compared to TKA without PPR regarding the EQ-VAS, the WOMAC total score and the WOMAC pain score 1 year after TKA?

RQ2: Could a treatment effect be observed for TKA with PPR compared to TKA without PPR regarding RTS of patients 1 year after TKA?

## Materials and methods

### Inclusion criteria

This study was a part of the research project MobilE-PRO within the MobilE-NET (Enabling participation by enabling MOBILity in older adults—Evidence-based health care research Network). This project evaluated TKA and THR patients with OA in routine care at a single high-volume hospital in Germany, certified as an arthroplasty center of maximum medical care (ClarCert®). The study was conducted between 5 August 2019 and 30 November 2020. 1146 patients from the hospital registry signed the consent to participate. 6 patients with other diagnoses than OA, 77 patients with data errors and 96 patients with a second surgery during the follow-up period were excluded. Hence, 967 patients were included in the study. 700 patients (499 THA, 201 TKA) answered the follow-up 1 year after surgery, resulting in a rate of return of 73%. Drop-out patients were analyzed with a binomial model with logit-link (Supplement Table 3). The results revealed no differences between patients with a follow-up compared to drop-out patients, regarding gender, age, insurance status, WOMAC total score, EQ-VAS, sport, ASA Score, BMI and lockdownstatus. From the 201 TKA patients, 36 patients with unicompartmental and 9 constrained implants were excluded because these implant types were considered to be not comparable. 156 patients remained for the final analyses, 74 patients received TKA with PPR and 82 patients without PPR. The study was approved by the ethics committee of Ludwig-Maximilians-Universität München (reference number: 18–274).

### Selection of patients for PPR and surgical technique

Patients with PPR were selected due to their intraoperational indication. PPR was chosen for patients with a high degree of cartilage damage (≥ III-IV according to ICRS, (Bittberg et al. [4]), especially in the area of the patella ridge and the lateral facet. Additionally, patients with patella maltracking, anterior knee pain, or inflammable knee disease were selected for PPR.

The implants were almost from one implant producer, with an all-poly-patella, and were cemented with an antibiotic-loaded bone cement form the same producer. The implantation was performed with a mechanical alignment and 90 degree-resection on the mechanical tibia- and femur axis with a rectangular extension and flexion gap. The patella was resected, resulting in at least 12 mm residual thickness and with avoiding offerstuffings.

For patellar resurfacing, the leg was fully extended, and the patella emerged at least 90 degrees. The soft tissue around the patella was then incised down to the insertion of the quadriceps and patellar tendons and the maximum thickness of the patella was determined by using a femur caliper to measure the most prominent anterior-to-posterior dimension. A 3.2 mm drill was used to drill the highest portion of the medial facet perpendicular to the articular surface approximately 12 mm deep centered on the medial sagittal ridge for proper medialization of the patella. A patella osteotomy guide was used with the stylus set for the desired amount of resection. After patella osteotomy a sizing guide helped to find the right size with no overhang centered over the drill hole.

All six involved surgeons proceeded according to one, standardized operation principle. The tibial horizontal cut was performed first, followed by the distal femoral cut. All operations were performed using the freehand technique without navigation or robotics. The alignment strategy was the adjusted mechanical alignment, in which the distal femoral angle was adjusted. The procedure of TKA is highly standardized, because the participating hospital is certified as an arthroplasty center of maximal medical care (ClarCert ®). All surgeons involved in the study were certified surgeons according to ClarCert ® certification criteria. Thus, each surgeon performed at least 50 endoprosthesis procedures per year.

### Patient reported outcomes (PROM) and sport

Health-related quality of life (HRQoL) was measured with generic and disease specific PROMs. The EQ-VAS served as the generic instrument and the WOMAC was selected for the disease specific measurement, specifically the pain score. The WOMAC total score was scaled from 0 to 100 to allow comparisons with other publications [25]. Patients answered the PROMs preoperatively as electronic PROMs (ePROM), and 1 year postoperatively either as ePROM or with paper and pencil. Sport habits were requested together with the PROMs and ePROMs questionnaires. Specific types of sport were asked for (cycling, walking, tennis, running, gym, skiing and other) with three levels of intensity (never, sometimes, regularly). (Supplement Table 4) These three levels of intensity were created as an ordinal variable preoperatively and postoperatively. The patient cohort was too small to include the specific types of sport, hence only the intensity was evaluated. RTS rates were calculated considering these intensities. Patients who were not changing their sport habits, for example doing any type of sport regular preoperatively and postoperatively, were defined as patients with the same intensity. Higher intensity was defined as patients who were increasing their sport habits, for example from doing any type of sport sometimes to regular. Patients with a reduction in their sport habits, for example from doing any type of sport regular to sometimes, were defined as less intense. RTS included uptake of sport by those who had not done so before. The overall RTS rate was calculated as the sum of patients with the same or higher intensity of sport, divided by the entire cohort, with and without PPR, respectively.

### Minimum clinical important difference (MCID) and minimum important change (MIC)

In addition to the statistical significance, the clinically relevant differences for the WOMAC total score were determined by Clement et al. [6] for TKA with anchor-based methods. Minimum clinical important difference (MCID) was defined as the difference between two groups, postoperatively, with 10 points for the WOMAC total score and 11 points for the WOMAC pain score [6]. The change between the preoperative and the postoperative score was determined as minimum important change (MIC) with 17 points for the WOMAC total score and 21 for the WOMAC pain score [6]. For the EQ-VAS the MCID was reported with 6.41 points and the MIC with 5.27 for TKA, equally with anchor-based methods [30].

### Statistical analyses

Due to the routinely collected data, there was no randomization of patients. Hence, a quasi-experiment with a Difference-in-Difference (DiD) approach with ordinary least squares (OLS) was applied, to evaluate the treatment effect of PPR. This method is suggested by Dimick and Ryan [10] to evaluate whether patients with and without an intervention had different outcomes. This approach compares the difference of outcomes before and after an intervention, for the intervention group and the control group. The intervention had an effect whether the difference of the differences (intervention to control group) was not zero. The aim of the statistical analyses was the determination of the causal effect of PPR on the WOMAC total score, the EQ-VAS and RTS. Initially developed for policy measures, this approach was already applied for medical registry data and clinical outcomes. [10]

In this study, the group with TKA and PPR was determined as the intervention group and patients with TKA without PPR were defined as the control group. The WOMAC total score, WOMAC pain score, EQ-VAS and RTS, as an ordinal variable with 3 intensities, were defined as the outcomes, measured at 2 time points (preoperatively and 1 year postoperatively). The parallel trend assumption of the DiD was regarded to be met, because all patients received TKA and would therefore develop similar trends for all outcomes, even without PPR. A p-value smaller than 0.05 was considered significant. The data collection was conducted with Microsoft Excel ® and the statistical analyses with R [16, 22, 28, 29, 31–33].

### Confounders

Since the study was evaluated with a DiD approach, a thorough selection of confounders was suggested by Zeldow and Hatfield [34]. All confounders were time-invariant and considered due to the possible associations with the outcomes (PROMs and RTS). The gender was included because more women suffer from OA than men and the levels of HRQoL may differ between genders. Furthermore, the age, Body Mass Index (BMI), ASA Score (American Society of Anesthesiologists Physical Status Classification System) and postoperative complications were analyzed, and the insurance status was included as a proxy for the socioeconomic status. The questions about postoperative complications were developed form physicians of the participating hospital. (Supplement Table 5).

Furthermore, the study was conducted during the COVID-19 pandemic. This was an external shock and violated the assumptions for DiD [10]. Hence, the lockdownstatus of patients, as evaluated in a previous study [24], was included. This lockdownstatus is a variable indicating whether a patient had the surgery before, during or after the first COVID-19 lockdown in Germany. Hence, circumstances related to this lockdown were controlled, to avoid a bias due to the COVID-19 pandemic.

### Sensitivity analyses

Due to the DiD approach with OLS the ordinal variable for RTS was treated as a metric variable. The logic behind this approach was the assumption that the transition between the levels (never, sometimes, regular) were, in reality, without boundaries for patients. Although, a generalized linear model with poisson distribution was calculated, to allow an analysis of this ordinal variable as a delimited variable. There were no changes in the main results (Supplement Table 6). All four outcomes (WOMAC total score, WOMAC pain score, EQ-VAS and RTS) were evaluated without any confounder and no significant result was found.

## Results

### Study cohort

Patient characteristics were similar with and without PPR, regarding age, BMI, postoperative complications and ASA scores. More women than men (43% versus 35%) and more patients with a non-statutory insurance received PPR. To mention that only 14% (22 patients) among the entire cohort were non-statutory insured (Table 1).

**Table 1:** Study cohort

Parameter	Entire cohort (N = 156)			PPR (N = 74)			No PPR (N = 82)		
	n	mean ± SD (%)	min | max	n	mean ± SD (%)	min | max	n	mean ± SD (%)	min | max
WOMAC total score preoperative	156	48.7 ± 18.2	5.0 | 95.4	74	50.3 ± 18.2	22.2 | 94.2	82	47.3 ± 18.1	6.7 | 95.4
WOMAC pain preoperative	156	9.8 ± 3.8	0.8 | 20.4	74	9.8 ± 3.9	0.8 | 20.4	82	9.8 ± 1.3	1.3 | 18.3
WOMAC total score postoperative	156	16.6 ± 19.6	0.0 | 95.4	74	14.1 ± 16.3	0.0 | 72.9	82	18.9 ± 22.1	0.0 | 95.4
WOMAC pain postoperative	156	2.9 ± 4.1	0.0 | 20.0	74	2.3 ± 3.3	0.0 | 12.5	82	3.4 ± 4.6	0.0 | 20
WOMAC change preoperative-postoperative	156	– 32.0 ± 21.4	– 87.5 | 35.4	74	– 36.2 ± 19.3	– 87.5 | 2.9	82	– 28.4 ± 22.6	– 69.2 | 35.4
WOMAC pain change preoperative-postoperative	156	– 7.0 ± 4.8	– 19.2 | 5.4	74	– 7.6 ± 4.5	– 19.2 | 3.3	82	– 6.4 ± 5.1	– 17.1 | 5.4
EQ VAS preoperative	156	56.9 ± 21.2	0 | 100	74	58.7 ± 20.8	0 | 100	82	55.2 ± 21.5	0 | 90
EQ VAS postoperative	156	71.9 ± 20.5	5 | 100	74	72.0 ± 20.0	5 | 100	82	71.9 ± 21.1	20 | 100
EQ VAS change preoperative-postoperative	156	15.0 ± 27.6	– 75 | 90	74	13.3 ± 28.6	– 75 | 90	82	16.7 ± 26.8	– 31 | 90
Sport preoperative	82	(53)		45	(61)		37	(45)	
Sport preoperative never	74	(47)		29	(39)		45	(55)	
Sport preoperative sometimes	10	(6)		6	(8)		4	(5)	
Sport activity preoperative regular	72	(46)		39	(53)		33	(40)	
Sport postoperative	102	(65)		50	(68)		52	(63)	
Sport postoperative never	54	(35)		24	(32)		30	(37)	
Sport postoperative sometimes	17	(11)		10	(14)		7	(9)	
Sport postoperative regular	85	(54)		40	(54)		45	(55)	
Age in years	156	71.4 ± 8.8	44.0 | 87.0	74	72.6 ± 8.5	44.0 | 87.0	82	70.2 ± 8.9	53.0 | 86.0
Gender male	61	(39)		26	(35)		35	(43)	
BMI	156	28.6 ± 5.0	19.6 | 44.8	74	28.5 ± 4.9	19.6 | 43.8	82	28.8 ± 5.1	20.9 | 44.8
Insurance status non-statutory	22	(14)		19	(26)		3	(4)	
Postoperative complications	28	(18)		13	(18)		15	(18)	
Postoperative complications surgery related ^1^	8	(5)		3	(4)		5	(6)	
Postoperative complications not surgery related ^1^	23	(15)		12	(16)		11	(13)	
ASA Score	156	2.1 ± 0.5	1 | 4	74	2.1 ± 0.5	1 | 3	82	2.1 ± 0.5	1 | 4
1	10	(6)		5	(6)		5	(6)	
2	118	(76)		56	(76)		62	(76)	
3/4	28	(18)		13	(18)		15	(18)	
Lockdownstatus									
Before first lockdown	79	(51)		42	(57)		37	(45)	
During first lockdown	13	(8)		7	(9)		6	(7)	
After first lockdown	64	(41)		25	(34)		39	(48)	

^1^multiple answers possible, *PPR* Primary Patella Resurfacing, *min* minimum, *max* maximum, *SD* standard deviation, mean = arithmetic mean

**Fig. 1:**
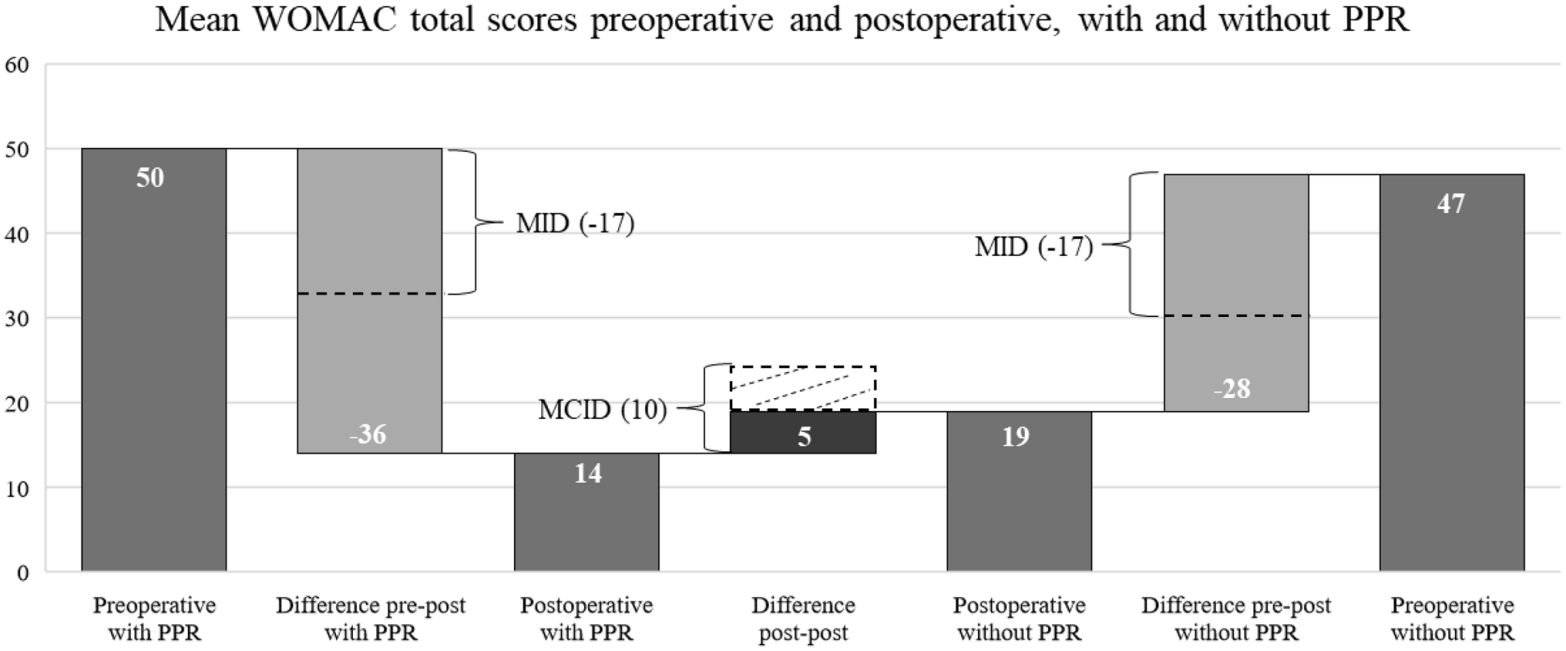
Mean WOMAC total scores

**Fig. 2:**
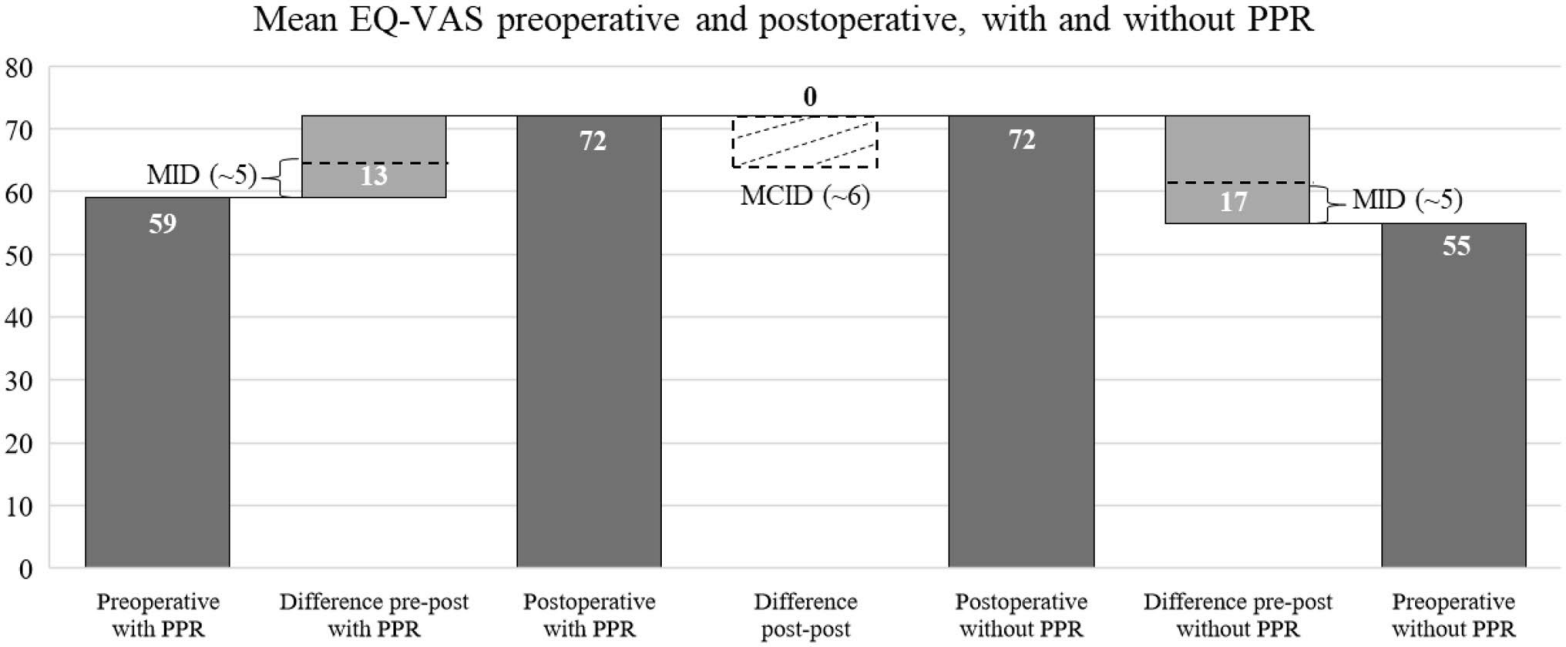
Mean EQ-VAS

The mean WOMAC total scores were postoperatively slightly better for patients with PPR (14.1 points versus 18.9 points without PPR), partly due to worse preoperative scores (50.3 with PPR versus 47.3 without PPR). The WOMAC pain scores were in the mean also slightly better for PPR patients (2.3 points versus 3.4 points without PPR), but with equal preoperative scores. The mean of the postoperative EQ-VAS was almost equal for patients with and without PPR (72.0 points with PPR versus 71.9 points without PPR). The MCID was not reached for the WOMAC total score, the WOMAC pain score and the EQ-VAS. However, the MID was reached generally for TKA for all PROMs, regardless of PPR (Table 1, Figs. 1 and 2).

Preoperatively, more patients stated to do sport with PPR (61% versus 45% without PPR), but postoperatively nearly the same proportion of patients stated to do sport (68% versus 63% without PPR). This indicated that patients without PPR improved their sport habits more than patients with PPR. To mention, that the entire cohort enhanced their sportive activities and returned to their sport habits, regardless of PPR. The majority of patients in both groups did sport regularly, preoperatively and postoperatively. Overall RTS rate was 94% for the entire cohort, for patients with PPR 93% and without PPR 95% (Table 1).

**Fig. 3:**
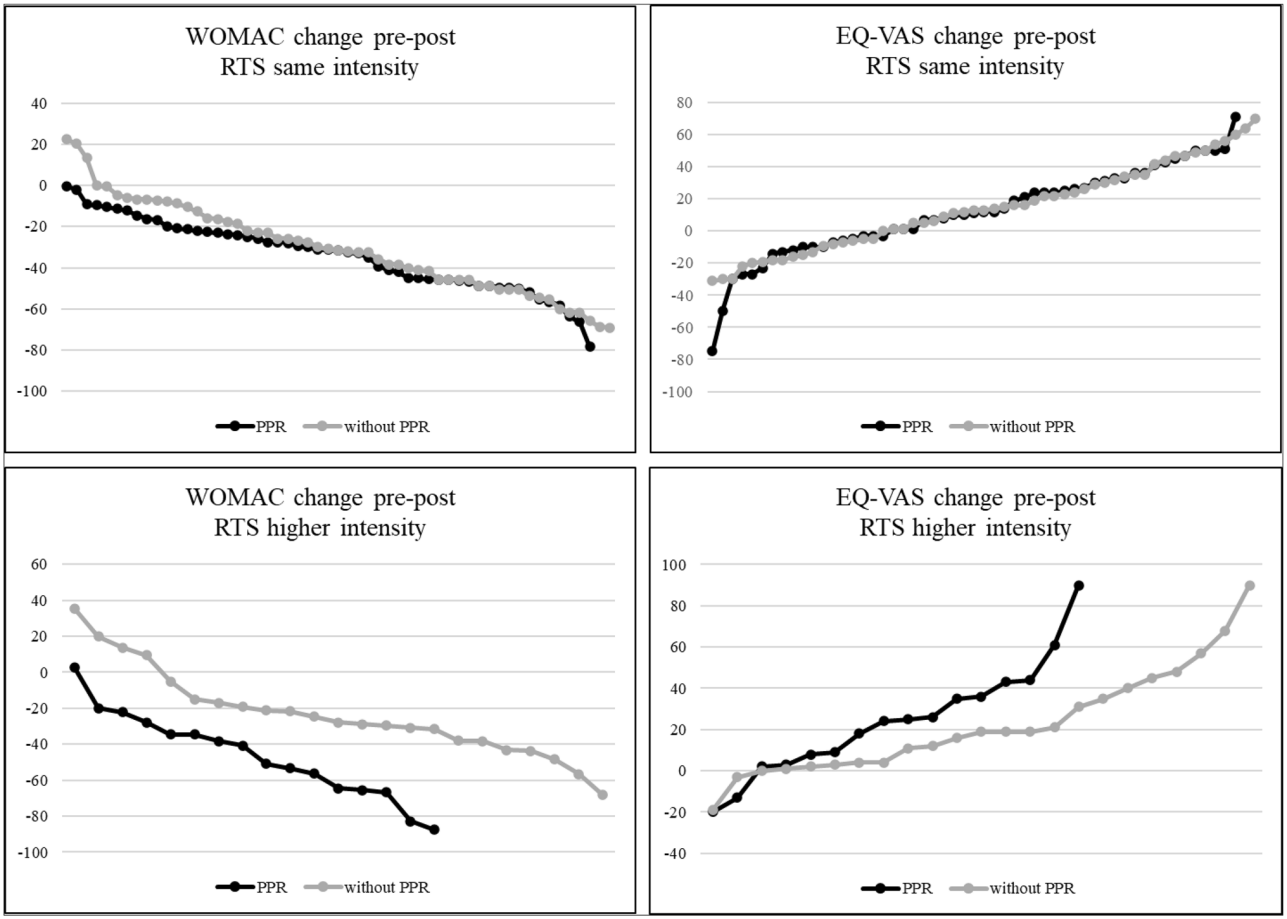
WOMAC and EQ-VAS change for RTS with the same and higher intensity

A distinguished analyzes of RTS with the same intensity, compared to RTS with higher intensity, indicated that PPR patients improved their generic and disease specific PROMs more than patients without PPR. However, there were almost no differences for patients with and without PPR for RTS with the same intensity (Fig. 3).

9 patients (5 with PPR, 4 without PPR) did less sport postoperatively compared to preoperatively. All these patients had improvements in the WOMAC total score (at least 26 points) and WOMAC pain score (at least 7 points). Although, 5 patients, out of these 9 patients, had lower EQ-VAS postoperatively and 1 patient reported complications, not related to surgery.

### Results PROMs and RTS

The results of the DiD estimations indicated no treatment effect for the EQ-VAS, the WOMAC total score, the WOMAC pain score and RTS. The preoperative HRQoL had the main effect on the EQ-VAS (16.83, p < 0.001), the WOMAC total score ( – 28.52, p < 0.001) and the WOMAC pain score ( – 6.34, p < 0.001). Furthermore, the gender had a slightly significant influence on all PROMs, where male gender indicated somewhat improved PROMs, compared to women. The EQ-VAS was additionally slightly influenced by postoperative complications (not related to surgery). RTS was slightly influenced by age ( – 0.03, p < 0.001) and BMI ( – 0.08, p < 0.001). With increasing age and BMI, patients were likely to do less sport, but with no effect of PPR (Table 2).

**Table 2:** Result treatment effect

Parameter	WOMAC total score			WOMAC pain		
	Coefficient	CI 95%	p-value	Coefficient	CI 95%	p-value
Treatment group	2.11	– 7.56 | 11.79	N.S.	– 0.34	– 2.34 | 1.67	N.S.
Post-treatment	– 28.52	– 37.32 | – 19.74	< 0.001 ***	– 6.34	– 8.16 | – 4.52	< 0.001 ***
Treatment effect	– 3.83	– 16.59 | 8.94	N.S.	– 0.25	– 2.89 | 2.40	N.S.
Age	0.04	– 0.36 | 0.44	N.S.	0.04	– 0.12 | 0.05	N.S.
Gender male	– 8.96	– 15.98 | -1.95	0.012 *	– 1.74	– 3.20 | – 0.29	0.019 *
ASA Score	5.79	– 1.75 | 13.33	N.S.	0.81	– 0.75 | 2.37	N.S.
Insurance status	– 0.40	– 9.42 | 8.62	N.S.	0.49	– 1.38 | 2.36	N.S.
Postoperative complications	5.28	– 3.71 | 14.27	N.S.	0.95	– 0.91 | 2.81	N.S.
BMI	– 0.22	– 0.97 | 0.54	N.S.	– 0.04	– 0.19 | 0.11	N.S.
Lockdownstatus	– 3.83	– 1.47 | 6.72	N.S.	0.44	– 0.41 | 1.28	N.S.
Adj. R^2^	0.367			0.377		

Parameter	EQ-VAS			Sport		
	Coefficient	CI 95%	p-value	Coefficient	CI 95%	p-value
Treatment group	1.53	– 8.06 | 11.11	N.S.	0.40	– 0.01 | 0.80	N.S.
Post-treatment	16.83	8.12 | 25.54	< 0.001 ***	0.29	– 0.70 | 0.66	N.S.
Treatment effect	– 3.48	– 16.13 | 9.17	N.S.	– 0.24	– 0.77 | 0.29	N.S.
Age	0.06	– 0.34 | 0.46	N.S.	– 0.03	– 0.05 | – 0.02	< 0.001 ***
Gender male	8.25	1.30 | 15.21	0.020 *	0.29	0.00 | 0.58	0.045 *
ASA Score	– 6.01	– 13.48 | 1.47	N.S.	– 0.01	– 0.30 | 0.32	N.S.
Insurance status	2.37	– 6.57 | 11.32	N.S.	0.19	– 0.18 | 0.57	N.S.
Postoperative complications	– 9.93	– 18.84 | – 1.03	0.029 *	– 0.26	– 0.63 | 0.11	N.S.
BMI	– 0.28	– 1.03 | 0.46	N.S.	– 0.08	– 0.11 | – 0.05	< 0.001 ***
Lockdownstatus	– 1.28	– 5.34 | 2.78	N.S.	– 0.05	– 0.21 | 0.12	N.S.
Adj. R^2^	0.162			0.266		

*CI* confidence interval, Significance levels * < 0.05, ** < 0.01, *** < 0.001

## Discussion

The main result of this study revealed no treatment effect for TKA with PPR, regarding PROMs and RTS. Although, the postoperative WOMAC pain score was descriptively slightly better and the improvement of the WOMAC total score was somewhat enhanced for patients with PPR, but without a statistically significant treatment effect and without reaching the MCID. The slightly more pain for patients without PPR, did not result in reduced RTS for patients 1 year postoperatively. Interestingly, patients without PPR did less sport preoperatively, but were equally active 1 year postoperatively. Additionally, patients with PPR and RTS with higher intensity had higher improvements of their generic and disease specific HRQoL. The main strength of this study was the simultaneous evaluation of PROMs and RTS. Moreover, the control- and intervention group were in fact comparable, with nearly the same means regarding patient characteristics. The routine data comprised patients treated by six different surgeons. This might be a strength, because different levels of experience of surgeons were included. Although, there was no randomization of surgeons, and hence a bias towards specific surgeons cannot be excluded. However, the participating hospital has highly standardized processes and almost the same manufacturer for the implants for all patients, which reduces the risk for biases towards specific surgeons.

The findings of this study were generally in line with previous studies on PROMs and PPR. Several randomized controlled trials (RCT) investigated whether PPR in TKA had a significant effect on a wide range of outcomes. A meta-analysis by Chen et al. [5] with 32 RCTs, between 1996 and 2020, recommended PPR. For at least a period of 5 years decreasing reoperation, better Knee Society Scores (KSS), enhanced function, and decreased noise were reported [5]. Although, short term reoperation, anterior knee pain (AKP) and patient reported outcomes (PROM), measured with the Oxford Knee Score (OKS), Osteoarthritis Outcome Score (KOOS), Visual Analog Scale (VAS) and satisfaction of patients were not different regarding PPR [5]. A most recent RCT from Deroche et al. [9] found no evidence for PPR with very low reoperation rates, but more pain for patients without PPR, 18 months postoperatively. Less patients reported AKP with PPR 1 year postoperatively [14]. PROMs, measured with the Western Ontario McMaster University Osteoarthritis Index (WOMAC), were not different for patients with PPR compared to patients without PPR, except the pain score for a follow-up period of 5 years [7]. Kaseb et al. [13] found 6 months postoperatively no differences in WOMAC Scores, AKP, KSS and pain scores. For isolated patellafemoral OA, Peng et al. [20] examined 7 eligible studies in a systematic review. Functional scores and physical activity were enhanced with patellafemoral replacement instead of TKA [20]. Ponzio et al. [21] found 2 years after surgery less improvement for preoperative physically active patients, for TKA patients in general. This study revealed again slightly higher pain of patients without PPR postoperatively, but it additionally discovered that these slightly higher pain did not hinder patients from doing their usual sport. RTS rates were almost equal for patients with and without PPR. Hence, PPR had no significantly measurable impact for patients, concerning their sport habits, generic and disease specific PROMs. Given the risks for PPR there seems to be no direct benefit for patients with PPR and hence no sensible cause to decide in favor of PPR. However, the reasons for these not measurable differences may be diverse. All patients with OA and TKA were included, without a distinguished analyses whether patients had patellofemoral OA and to which degree. This may be the reason why no differences were found because patients with patellofemoral OA needed PPR. There was maybe no choice for or against PPR for those patients. This may violate the parallel trend assumption for the DiD approach. Further research is necessary, with a distinguished analyses of patellofemoral OA patients versus patients with no patellofemoral OA, to clarify this issue. Another reason why this study found no differences might be the measurement of pain. The WOMAC pain score is an approved and often applied instrument for TKA, but it does not specifically measure the AKP. Although, AKP is found to be decreased for patients with PPR [11]. Further research should include measurements for AKP to evaluate possible differences for patients. The cause for the less preoperative sport for patients without PPR may be the insurance status. Previous literature revealed that slightly more patients follow physical activity recommendations with a private insurance [35]. This might partly explain the lower preoperative sport levels for patients without PPR. Another important factor was the time to follow-up. Previous literature evaluated with data from the large Swedish hip register, that the results of PROMs a 1 year after surgery were very similar to PROMs with a 6-year follow-up period [3]. Hence, this study evaluated PROMs and leisure sport habits 1 year after surgery. However, maybe 1 year was too short, or too long to find significant results. To mention, that a shorter period may cause a bias, because patients need maybe more time to recover, and a longer period may cause the risk for other complications, not related to surgery. An interesting side result of this study indicated that complications, not related to surgery, were associated with the EQ-VAS. Hence, whether further research is evaluating longer time periods, a thorough analyses of complications is recommended [18].

This study has several limitations. The questionnaire about sport habits has not been validated psychometrically. This could result in biases, due to misunderstandings about the definition of different types of sport. Furthermore, the time period of doing sport was not determined, for example patients may understand the question in general for several years of their life, or for the last week. These misunderstandings may blur or bias the results. The intensity of sport was distinguished only between three intensity levels and this ordinal variable was applied as a metric variable. Sensitivity analyses found no differences in the main results. This may be too superficially to find differences between groups. Generalizability is limited, because the patient cohort was rather small with less than 100 patients in each group and the study took place in one hospital in Germany. Hence, the statistical power might not be high enough to find significant results. The study cohort was thoroughly selected, with patients who had OA and a primary TKA. This might not be specific enough to find significant differences. Further research might collect a higher number of patients and might be able to build other sub-groups. Furthermore, the impact of COVID-19 was evaluated to the best of our knowledge, but a bias could not be entirely excluded. The results indicated that patients were treated similar regarding PPR, before, during and after the first COVID-19 lockdown in Germany. The external shock seemed to have no important impact on the surgery of TKA.

## Conclusions

Comparing TKA patients with PPR and TKA patients without PPR, this observational study found no treatment effect both for PROMs and RTS. The WOMAC total score and the WOMAC pain score were slightly better for patients with PPR, but without reaching clinical relevance. There seemed to be a trend towards higher improvements for the WOMAC total score and the EQ-VAS, for patients with increasing sportive activities with PPR compared to patients without PPR. Approximately 94% of all patients returned to their usual sport habits 1 year after surgery, regardless of PPR. Hence, the evidence about PPR in TKA remains vague. There seems to be no measurable benefit for PPR, considering PROMs and RTS. Regarding these two endpoint categories, hence no clear treatment recommendation can be given.

## Supplementary Information

Below is the link to the electronic supplementary material.

Supplementary file1 (PDF 40 KB)

## Data Availability

Data is not publicly available, due to privacy issues.
