# Supplementary material for: The impact of primary patella resurfacing on health-related quality of life outcomes and return to sport in total knee arthroplasty (TKA)
Source: Arch Orthop Trauma Surg. 2023 Jun 8;143(11):6731–40. doi: 10.1007/s00402-023-04930-x (PMC10248965; doi:10.1007/s00402-023-04930-x)
Supplement: Supplementary file 1 — Supplementary file1 (PDF 40 KB) [file 402_2023_4930_MOESM1_ESM.pdf]

Supplement

Table 3: Logisitic regression drop outs

Parameter	Estimate (log-odds)	p-value
Gender male	0.075	0.737
Insurance status	0.035	0.916
Age	-0.005	0.698
Lockdownstatus	-0.132	0.251
WOMAC total score preoperative	-0.000	0.979
EQ-VAS preoperative	0.000	0.980
Sport preoperative	0.006	0.980
ASA Score	-0.041	0.857
BMI	0.028	0.180

Logisitic regression with log-link function, p-value with z-statistic.

The logistic regression indicates no differences between the group of patients, who participated in the follow-up and who did not, because all p-values were insignificant.

Table 4: Sport questions

**Question**	**Answer**
Are you doing sport?	yes/no
Cycling	regular, sometimes, never
Walking/Hiking	regular, sometimes, never
Tennis	regular, sometimes, never
Skiing	regular, sometimes, never
Gym	regular, sometimes, never
Running	regular, sometimes, never
Other	regular, sometimes, never

Questions were asked preoperatively and postoperatively (free translation from German).
For each answer the patients had to tick a box.

Table 5: Questions about postoperative complications

**Questions related to surgery**	**Answer**
Did you experience complications or severe health problems after discharge from the hospital?	yes/no
Reoperation of the endoprosthesis?	yes/no
Secondary bleeding with severe bruise?	yes/no
Luxation (Dislocation) of the endoprosthesis?	yes/no
Infection of the endoprosthesis?	yes/no
Loosening of the endoprosthesis?	yes/no

**Questions not related to surgery**	**Answer**
Thrombosis?	yes/no
Pulmonary embolism?	yes/no
Heart attack?	yes/no
Other severe illness?	yes/no

Questions were asked postoperatively (free translation from German). For each answer the patients had to tick a box.

Table 6: Sensitivity analyses sport

Parameter	Physical activity		
	Odds ratio	CI 95% [1]	p-value [2]
Treatment group	2.98	0.92 | 9.69	N.S.
Post-treatment	2.62	0.92 | 7.49	N.S.
Treatment effect	0.55	0.11 | 2.69	N.S.
Age	0.91	0.86 | 0.96	< 0.001 ***
Gender male	2.02	0.81 | 5.08	N.S.
ASA Score	1.01	0.37 | 2.77	N.S.
Insurance status	4.71	1.19 | 18.73	0.023 *
Postoperative complications	0.40	0.13 | 0.87	N.S.
BMI	0.78	0.70 | 0.87	< 0.001 ***
Adj. R^2	0.439 [3]		

[1] Wald statistics; [2] z-statistics (chis-squared); [3] Nagelkerke (1991); CI = confidence interval
